# Towards Understanding Neurodegenerative Diseases: Insights from *Caenorhabditis elegans*

**DOI:** 10.3390/ijms25010443

**Published:** 2023-12-28

**Authors:** Yingjie Wu, Yining Chen, Xiaochun Yu, Minxing Zhang, Zhaoyu Li

**Affiliations:** Queensland Brain Institute, The University of Queensland, Brisbane, QLD 4072, Australia; uqywu29@uq.edu.au (Y.W.); yining.chen@uq.edu.au (Y.C.); xiaochun.yu@uq.net.au (X.Y.); minxing.zhang@uq.net.au (M.Z.)

**Keywords:** *C. elegans*, gain-of-toxicity, SOD1, TDP-43, FUS, C9ORF72, polyQ, tau, amyloid β1-42, α-synuclein

## Abstract

The elevated occurrence of debilitating neurodegenerative disorders, such as amyotrophic lateral sclerosis (ALS), Huntington’s disease (HD), Alzheimer’s disease (AD), Parkinson’s disease (PD) and Machado–Joseph disease (MJD), demands urgent disease-modifying therapeutics. Owing to the evolutionarily conserved molecular signalling pathways with mammalian species and facile genetic manipulation, the nematode *Caenorhabditis elegans* (*C. elegans*) emerges as a powerful and manipulative model system for mechanistic insights into neurodegenerative diseases. Herein, we review several representative *C. elegans* models established for five common neurodegenerative diseases, which closely simulate disease phenotypes specifically in the gain-of-function aspect. We exemplify applications of high-throughput genetic and drug screenings to illustrate the potential of *C. elegans* to probe novel therapeutic targets. This review highlights the utility of *C. elegans* as a comprehensive and versatile platform for the dissection of neurodegenerative diseases at the molecular level.

## 1. Introduction

The prolonged average human lifespan is accompanied by an increased incidence of ageing-associated neurodegenerative disorders, including amyotrophic lateral sclerosis (ALS), Huntington’s disease (HD), Alzheimer’s disease (AD), Parkinson’s disease (PD), Machado–Joseph disease (MJD) and other neurological diseases. The growing economic and social burdens imposed by these diseases on global healthcare systems necessitate an urgent solution to diminish their impact. Unfortunately, there have not yet been any effective treatments to unequivocally stop or slow down the disease progression. The ambiguity in current knowledge about disease-causing molecular mechanisms remains an obstacle in developing novel drugs for the diseases.

Since its inception as an experimental organism in the 1970s [1], *Caenorhabditis elegans* (*C. elegans*) has rapidly emerged as a simple and cost-effective model system for human diseases. The worm is a small (~1 mm), free-living and self-producing nematode feeding on a bacterial diet of different species [1]. It has been widely utilised as a paradigm for studies of neurodegenerative disorders, owing to its short life cycle of around 2 to 3 weeks, simple laboratory handling and transparent nature, facilitating the live observation of fluorescence-tagged neurons [2]. Its explicitly mapped network of 302 neurons provides a direct and reliable approach for precise neuronal tracking and analyses [3]. The high genetic and functional conservation between the *C. elegans* genome and the one of mammals [4] enables comparative studies of specific cellular mechanisms and molecular pathways. From a genetic point of view, *C. elegans* is amenable to high-throughput genetic and drug screens, which provides a unique opportunity to explore molecular mechanisms and therapeutic options for neurodegenerative diseases. 

**Figure 1:**
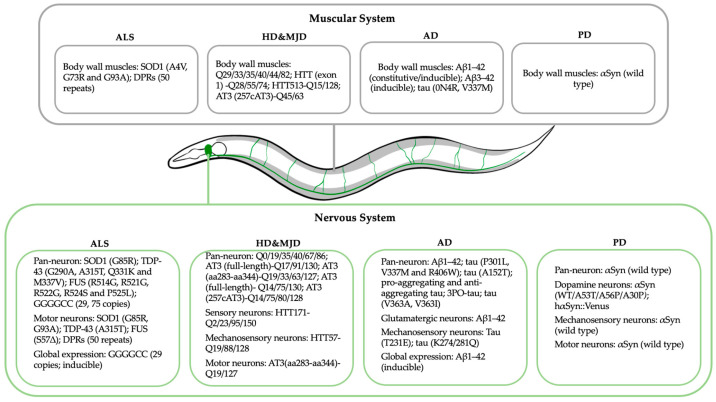
A simplified anatomical sketch of *C. elegans* denoting tissues of transgene expression applied in the reviewed disease models. Regions of expression are separated into two organ systems with 4 sub-divisions of specific neurodegenerative diseases. Green: nervous system; grey: muscular system.

In this review, we provide an up-to-date outline of studies that utilise *C. elegans* as a model organism to investigate the cellular and molecular basis of neurodegenerative diseases. We mainly focus on the currently existing “gain-of-function” models, in the context of five common neurodegenerative diseases, amyotrophic lateral sclerosis (ALS), Huntington’s disease (HD), Alzheimer’s disease (AD), Parkinson’s disease (PD) and Machado–Joseph disease (MJD), for which *C. elegans* models have been well established. A graphic illustration of the transgene expression in relation to different disease models is depicted in Figure 1. 

## 2. Amyotrophic Lateral Sclerosis (ALS)

ALS is a lethal motor neuron disease characterised by the selective and gradual loss of motor neurons in the spinal, bulbar and cortical regions [5]. The vast majority of ALS cases are sporadic, while 5–10% of patients exhibit apparent autosomal dominant inheritance [5,6]. Several causative genes have been linked to familial ALS, including Cu/Zn-binding superoxide dismutase (*SOD1*), TAR DNA-binding protein (*TDP-43*), fused in sarcoma (*FUS*) and the chromosome 9 opening reading frame 72 (*C9ORF72*) [7].

### 2.1. Cu/Zn-Binding Superoxide Dismutase (SOD1) Models

*SOD1* was first identified as a causative gene of ALS in 1993 [8]. It functions as an antioxidant catalyst for the conversion of superoxide radicals into dioxygen and hydrogen peroxide, essentially preventing superoxide from damaging the cell [9]. To date, over 170 missense point mutations in *SOD1* have been discovered, accounting for 10–20% of familial ALS cases [9,10]. Although the exact molecular mechanism of SOD1 protein-related toxicity has not yet been delineated, increasing evidence supports that mutated SOD1 exerts its cytotoxic effects in a gain-of-function manner, causing aggregation, mitochondrial dysfunction, oxidative stress elevation and proteostasis disruption [11,12].

The gain-of-toxicity effects have been observed in transgenic *C. elegans* by introducing human SOD1 mutants. The overexpression of human SOD1 (G93A) in *C. elegans* motor neurons led to prominent SOD1 aggregates, axon guidance failure and motor defects [13,14]. Similarly, worms with the pan-neuronal expression of human SOD1 (G85R) displayed insoluble SOD1 aggregates, a reduced axonal size and number and significant locomotory impairment [15,16]. When overexpressing disease-associated SOD1 mutations (A4V, G73R and G93A) in *C. elegans* body wall muscles, it yielded similar gain-of-toxicity phenotypes, manifesting as the presence of SOD1 aggregates and severe appearance and locomotion anomalies upon exposure to paraquat-induced oxidative stress compared to a control strain [17]. In general, these studies have managed to recapitulate some of the characteristic clinical phenotypes of ALS, such as the progressive loss of motor capabilities, presence of toxic protein aggregates and axonal abnormalities [18].

The above models in different tissues have greatly facilitated genetic and drug screenings related to SOD1 toxicity. In the mammalian system, SOD1 neurotoxicity has been linked to the proteostasis network. The upregulation of the ubiquitin-proteosome pathway or autophagy activities effectively mitigates SOD1 toxicity [19]. A genome-wide RNA interference (RNAi) screen using the SOD1 (G93A) model in *C. elegans* corroborated the protective role of the proteostasis network in suppressing SOD1 toxicity and identified 63 genetic modifiers that were efficient in alleviating SOD1 aggregation. These modifiers incorporated different aspects of the proteostasis network from the chaperone system and ubiquitin-proteosome pathway to autophagy [20]. In another model, TorsinA, an ER protein acting in a chaperone-like fashion, attenuated SOD1 (G85R)-induced ER stress, promoted the proteasomal degradation of mutant SOD1 protein and rescued behavioural defects [21]. Interestingly, genes regulating ageing have also been identified to modulate SOD1 toxicity. The overexpression of *daf-16* alleviated aggregates formation and reversed the paralytic phenotype elicited by SOD1 mutations. Consistently, metformin, a lifespan extension drug, showed protective effects against SOD1-induced cytotoxicity. It significantly increased the lifespan and mitigated SOD1-induced locomotor dysfunctions, partially relying on a *daf-16*-dependent pathway [22]. Subsequently, metformin has recently entered a phase 2 clinical trial to examine its safety and efficacy in ALS patients [23].

### 2.2. TAR DNA-Binding Protein (TDP-43) Models

Mutations in *TDP-43* account for approximately 3% of familial ALS cases [24]. TDP-43 is a ubiquitously expressed DNA- and RNA-binding protein of 43 kDa that regulates transcription and alternative mRNA splicing and RNA stability [25]. In ALS patients, the sequestration and redistribution of phosphorylated TDP-43 proteins into intracytoplasmic ubiquitinated inclusions, accompanied by a significant depletion in natural nuclear TDP-43, were discovered in their brain samples [25,26,27]. A gain-of-toxicity from nuclear TDP-43 mislocalisation to cytosolic inclusions has been reported to contribute to TDP43 proteinopathy [25,28].

The pan-neuronal expression of ALS-linked human TDP-43 mutants (G290A, A315T, Q331K, M337V) elicited neurotoxicity in *C. elegans*. Worms exhibited distinct neurotoxic features including motor dysfunction, compromised longevity and solid inclusions with phosphorylated protein aggregates, analogous to the hallmarks of TDP-43 proteinopathy in humans [28,29]. When expressed in motor neurons alone, TDP-43 (A315T) caused the progressive deterioration of locomotor function, cytoplasmic insoluble aggregates and motor neuron degeneration, which resembled the cellular phenotypes of human ALS [30].

Phosphorylation has been identified to play an important role in TDP-43 toxicity in *C. elegans*. Using a *C. elegans* model, Liachko et al. [29] located the phosphorylation site at serine residues 409/410 (s409/410), as a main driving factor for the higher toxicity of mutant TDP-43 (G29A, M337V). In addition, a potent phosphatase, calcineurin, was recognised for its precise dephosphorylation at the s409/410 sites. The genetic inhibition of this phosphatase in *C. elegans* profoundly promotes phosphorylated accumulation and aggravates motor deficits [31]. Another drug screen has revealed an alternative potent drug candidate for its neuroprotective effects in treating TPD-43 mutant-caused neurotoxicity that resembles familial ALS characteristics in *C. elegans*. α-Methyl-α-phenylsuccinimide (MPS), an active metabolite of a widely used anti-epileptic drug, ethosuximide, rescued the locomotor deficits and extended the lifespan in the TDP-43 (A315T) model [32]. This effect was mainly mediated through the DAF-16-dependent insulin-like pathway, indicating the importance of the ageing pathway in relation to treating TDP-43 neurotoxicity [32]. These studies exemplify the practicability and robustness of the *C. elegans* model system for the high-throughput drug discovery of new drug candidates.

### 2.3. Fused in Sarcoma (FUS) Models

About 4% of familial ALS cases are attributed to mutations in *FUS*, a gene encoding DNA- and RNA-binding proteins that regulate DNA damage, RNA transcription, splicing and transport [33,34]. Similar to TDP-43, the proteinopathy of mutant FUS proteins is characterised by the cytosolic accumulation of toxic FUS aggregates alongside a loss of wild-type [35] proteins, dysfunctional mRNA metabolism and motor neuron degeneration [36,37].

The overexpression of human FUS mutations (R514G, R521G, R522G, R524S and P525L) pan-neuronally in *C. elegans* showed characteristic neuropathological changes, such as cytosolic aggregates, a gradual decline in locomotor activities and a reduced lifespan. The severity of each mutant corresponded to the level of clinical severity of each one in humans and failed to be restored by the WT FUS protein, indicating gain-of-function toxicity [35]. A consistent phenotype was observed in another study conducted by Vaccaro et al. [30], where they introduced full-length FUS variant S57∆ in *C. elegans* motor neurons. Labarre A [38] engineered a single-copy human FUS mutant model in motor neurons, which provoked a similar gain-of-toxicity phenotype, manifesting as progressive locomotory defects and destructive neuromuscular junctions. Prior studies have suggested a link between FUS toxicity and autophagy. For further investigation, Baskoylu et al. [39] introduced disease-causing mutations (R524S, P525L) into *C. elegans* FUS orthologue *fust-1*. The study revealed that the neurotoxicity of *fust-1* was partially due to the disturbance in autophagy following the loss of *fust-1*, highlighting possible cellular mechanisms of FUS proteinopathy [39]. Taken together, these models closely mimic the clinical features of mutant FUS-related ALS cases and provide valuable insights into the cellular mechanisms and pathogenesis of the disease.

### 2.4. Chromosome 9 Open Reading Frame 72 (C9ORF72) Models

Hexaneulotide (GGGGCC) repeat expansions within a non-coding region of the *C9ORF72* gene have been implicated to be responsible for 10–40% of familial cases, making this the unprecedently most frequent ALS-causing gene [40,41,42]. C9ORF72 proteins play a role in the regulation of intracellular endolysosome trafficking in the autophagy-lysosome pathway [43]. Typically, more than 30 hexanucleotide repeats is considered etiopathogenetic, although, in some ALS cases, the repeat counts can reach hundreds to thousands [42,44].

The overexpression of human C9ORF72 in *C. elegans*, consisting of 29 hexanucleotide repeats, either globally or pan-neuronally, causes a severe age-dependent decline in motility in parallel to a shortened lifespan [45]. This finding was further corroborated by a separate study, where worms expressing 75 GGGGCC repeats pan-neuronally developed a shortened lifespan, locomotor defects and distinct dipeptide repeat (DPRs) protein aggregates [46].

Although how exactly C9ORF72 confers toxicity remains enigmatic, a combination of loss-of-function and gain-of-function has been speculated [41,47]. Loss-of-function toxicity is a result of the perturbed regulation of normal gene expression, which ultimately leads to C9ORF72 haploinsufficiency [47]. In terms of gain-of-function toxicity, the leading theory is based on repeat-associated non-AUG (RAN) translation, translating sense and antisense transcripts containing GGGGCC repeats and producing five toxic dipeptide repeat (DPRs) proteins with the propensity to aggregate intracellularly [48]. The five DPRs translated from GGGGCC repeats include poly-glycine-alanine (GA), poly-glycine-proline (GP), poly-glycine-arginine (GR) in the sense direction and poly-proline-arginine (PR) and poly-proline-alanine (PA) in the antisense direction [47]. Several studies have reported that arginine-containing dipeptides PR and GR possess the highest toxicity. Worms expressing 50 repeats of PR or GR in either muscle or motor neurons developed an age-dependent paralytic pattern and stunted growth [49]. It is noted that the nuclear localisation of the peptide is required to exert toxic effects [49]. On this basis, Snoznik, et al. [50] performed a forward genetic screen and identified *spop-1*, an orthologue for human SPOP (a conserved nuclear E3 ubiquitin ligase adaptor protein), responsible for the neurotoxicity of PR50 and GR50. The inhibition of *spop-1* significantly improved the abnormal behavioural phenotypes in worms, presenting a potential druggable target for the alleviation the neurotoxicity of arginine-related dipeptides [50].

## 3. Polyglutamine (polyQ) Repeat Diseases

The abnormal expansion of CAG trinucleotide repeats in the coding regions of separate genes encoding polyglutamine (polyQ) tracts in a RAN translation fashion is the genetic cause of at least nine neurodegenerative disorders, among which Huntington’s disease (HD) and spinocerebellar ataxias (SCAs) represent the two most frequent forms [51]. Although the affected genes in different polyQ disorders are unrelated, all diseases share a common phenotypic feature that is slow and progressive, accompanied by a pathological threshold of polyQ length ranging from around 21 to over 100 for complete penetrance [51]. Proteins containing polyQ expansions are prone to misfolding and aggregation, and it is widely accepted by the current literature that polyQ aggregation may involve a gain-of-toxicity from the expanded polyglutamine repeats [52,53,54]. Intriguingly, a recent finding uncovered the aberrant accumulation of novel repeat peptide proteins produced through RAN translation of the CAG repeats, polyalanine, polyserine, polyleucine and polycysteine in HD human brains. This implies an uncharacterised pathogenetic pathway contributing to the neurotoxicity of CAG-repeat-related diseases [55].

### 3.1. Huntington’s Disease (HD) Models

HD is a dominantly inherited disorder that is monogenic, rare and fatal, with currently no disease-modifying treatment available. It is genetically caused by an elongated CAG repeat in exon 1 of the Huntingtin (*HTT*) gene that encodes an expanded polyQ stretch [56]. In normal populations, the number of CAG repeats is equal to or below 35, while in patients with HD, the disease is fully penetrant when the length of repeats exceeds 40 [57].

Clinical manifestations of HD include the progressive loss of motor control, such as chorea and incoordination, cognitive impairment and neuropsychiatric disorders [57]. A prominent reduction in striatal volume and atrophy of the caudate nucleus and putamen are the core neuropathological changes associated with HD [58]. Even though there is wide expression of HTT in human brains, GABAergic medium spiny neurons of the striatum suffer a strikingly selective vulnerability, subsequently subjecting them to neuronal dysfunction and cell death [59]. A hallmark pathological feature of HD is the deposit of intranuclear and cytoplasmic aggregates, with previous evidence found in post-mortem human HD brains, transgenic mouse models and in vitro cell culture models [60,61]. The exact physiological role of misfolded HTT is unclear; however, it is hypothesised that the expanded polyQ strand confers a toxic gain-of-function that results in neurodegeneration and the development of HD symptoms [58].

The absence of an *HTT* orthologue in *C. elegans* does not prevent it from becoming a suitable model organism for the investigation of the underlying mechanisms of neurotoxicity driven by polyQ. Several transgenic *C. elegans* models have been established to enable the expression of polyQ with varying lengths fused to fluorescent marker proteins in different groups of neurons—for example, in ASH sensory neurons of *C. elegans* under the control of the *osm-10* promoter [62]. The results demonstrated in this study are in consistency with the findings in human HD, indicating that the age of onset and disease severity are polyQ-length-dependent, and reveal a certain threshold of polyQ expansions for the appearance of mutant HTT aggregates [62]. The overexpression of HTT171-Q150 in ASH sensory neurons led to nose touch defects before the occurrence of major aggregation, indicating that cellular dysfunction mediated by mutant HTT might precede protein aggregation [62]. Another *C. elegans* model used the *mec-3* promoter to express mutant HTT in touch receptor neurons, where the perinuclear formation of aggregates along with axonal abnormalities were identified in both young and old adult animals [63]. No cell death was observed in this study, which might be attributed to the lack of intranuclear aggregate formation [63]. Furthermore, the pan-neuronal expression of polyQ in *C. elegans* was examined using the *rgef-1* promoter [64]. Behavioural assays reflected a significant correlation between the polyQ repeat size and neuronal dysfunction, and a pathogenic threshold of more than 40 glutamines was required for the formation of insoluble aggregates [64].

In addition to expressing mutant HTT or polyQ in the nervous system, there are muscle-specific *C. elegans* models, in which polyQ expression is confined to body wall muscle cells. Disease-length polyQ expressed in these cells under the control of the promoter *unc-54* caused reduced motility and a shortened lifespan compared to WT animals, and polyQ aggregation and toxicity were shown to increase with age [65,66]. According to the fluorescence distribution in muscle cells expressing polyQ, 35–40 glutamine residues was considered a threshold for aggregation and neuronal dysfunction [66]. Mutations in *age-1*, which could prolong the lifespan of *C. elegans* via an insulin-like pathway, contributed to the delayed onset of motility defects and polyQ aggregation [66]. Moreover, the overexpression of ubiquitin was found to alleviate the toxic effects associated with HTT-Q55 [67].

Owing to the facile genetics of *C. elegans*, forward and reverse genetic screens have been largely employed in *C. elegans* models to identify gene mutations that are of interest regarding polyQ toxicity. Using a previously described *C. elegans* model [62], genetic screens were conducted aiming to identify protective proteins against the toxic effects of polyQ, consequently giving rise to the discovery of the *polyQenhancer-1* (*pqe-1*) gene [68]. Mutations in *pqe-1* led to the enhancement of neurotoxicity in ASH sensory neurons, and neurodegeneration was exacerbated as the animals aged [68]. Other studies performed genome-wide RNAi screens in transgenic *C. elegans* models, which identified 88 genetic suppressors of polyQ aggregation and 23 of toxicity [20], as well as 49 modifiers of polyQ-mediated neuronal dysfunction that had been found previously in HD mice models [69]. Moreover, by performing a mutagenesis screen in a *C. elegans* model expressing Q40, a novel modifier of aggregation *moag-4* has been identified [70]. The inactivation of *moag-4* was shown to suppress polyQ aggregation in transgenic animals. Notably, MOAG-4 is highly conserved. Human orthologues SERF1A and SERF2 have also been shown to modulate polyQ aggregation and toxicity [70]. These results have further confirmed the genetic intersection between the nematode and mammals and therefore the feasibility of using *C. elegans* as models to interpret human HD.

A more recent study examined the toxic effects of all six repeat peptide products of CAG-related RAN translation, including polyglutamine in *C. elegans*, and reported polyleucine to convey the strongest toxicity, which caused the most penetrant phenotype of stunted growth and defective motility in worms [71]. This result corroborated the previous finding in HD human brains that an alternative mechanism might be responsible for the neurotoxicity of CAG-repeat-related neurodegenerative diseases other than the conventionally thought polyQ repeats [71].

### 3.2. Machado–Joseph Disease (MJD) Models

Spinocerebellar ataxia type 3 (SCA3), also referred to as Machado–Joseph disease (MJD), is a dominantly inherited neurodegenerative disorder that represents the most frequent form of SCAs worldwide [72,73,74]. MJD is caused by an abnormally expanded CAG repeat in exon 10 of the *ATXN3* gene [75]. The expansion of CAG repeats in individuals affected by MJD usually ranges from 60 to 87, while in healthy populations, it does not exceed 44 [76]. The age of onset of MJD is inversely proportional to the size of trinucleotide repeats, and the disease severity increases with the repeat length [72].

Clinically, MJD leads to progressive ataxia and pyramidal signs, accompanied by a wide array of symptoms such as amyotrophy, gait imbalance, ophthalmoplegia, speech difficulties and dysphagia [77]. Neuropathological findings of MJD are highlighted by prominent neuronal loss and the atrophy of brain structures, including the cerebellum, pons and basal ganglia [78]. Similar to HD, the accumulation of intranuclear and cytoplasmic aggregates is a common feature of MJD, as evidenced in human brain, transgenic animal and cell line studies [79,80].

In *C. elegans*, full-length and truncated ATXN3 with varying lengths of glutamines was expressed pan-neuronally under the control of the *unc-119* promoter, which caused motility deficits and neuronal dysfunction including an impaired ubiquitin-proteasome system (UPS), disrupted synaptic transmission and compromised neuronal processes [81]. It was reported in another model that intranuclear and cytoplasmic mutant ATXN3 aggregates accumulated in a polyQ-length-dependent manner in vivo, and protein aggregation followed a cell-type-specific pattern in the nervous system of *C. elegans*, where immobile aggregates were detected in ventral and dorsal nerve cord neurons but rarely in lateral interneurons [82]. Significantly reduced motility was also observed in animals in the presence of aggregates compared to the control group, suggesting a direct correlation between mutant ATXN3 aggregation and neuronal dysfunction [82]. Moreover, it has been found that the ageing-related transcription factors DAF-16 and heat-shock factor 1 (HSF-1) play a protective role against mutant ATXN3 pathogenesis [82]. A more recent muscle-specific *C. elegans* MJD model presents similar results that the aggregation and neurotoxicity driven by a C-terminal fragment of ATXN3 are dependent on the polyQ length [83]. Interestingly, the study found that ageing is not necessarily involved in the exacerbation of polyQ aggregation and toxicity.

A large-scale RNAi screen performed in a *C. elegans* transgenic model expressing mutant *ATXN3* gene led to the identification of a transcription-factor-coding gene *fkh-2/FOXG1*, which rescued the mutant ATXN3-induced motility defect, shortened lifespan and neurodegeneration when overexpressed [84]. In another *C. elegans* MJD model, the efficacy of befiradol was tested, which is an agonist specifically targeting the serotonin 5-HT_1A_ receptor, and both acute and chronic treatment resulted in a reduction in mutant ATXN3 aggregation [85].

## 4. Alzheimer’s Disease (AD)

Alzheimer’s disease (AD) is a chronic neurological disorder and a classic manifestation of dementia with an increased incidence with age [86]. AD affects diverse regions of the brain, including the hippocampus, temporal lobe, frontal lobe and limbic system [87]. The physiological consequences of AD encompass a broad spectrum of dysfunctions, such as memory loss, cognitive impairment and disturbances in consciousness [88]. Aggregation is a hallmark of AD that is believed to cause neural dysfunction and, ultimately, neuronal death [89].

Numerous genes are associated with AD pathology, namely the Apolipoprotein E (*APOE*), Microtubule-Associated Protein Tau (*MAPT*) and Amyloid-β Precursor Protein (*APP*) genes [89]. Mutations in the *APP* and *APOE* genes result in the elevated accumulation of Amyloid-β plaques between neurons, which further disrupts neuronal function and is recognised as an established hallmark of AD pathogenesis [89,90]. Additionally, AD is characterised by the presence of intracellular hyperphosphorylated tau aggregates, which form neurofibrillary tangles that hinder communication between brain cells [89]. The amyloid plaques and fibrillary tangles increase the production of toxic reactive oxygen species (ROS) and impair normal cellular machineries such as autophagy and mitochondrial function, which eventually contribute to cell death [91,92].

### 4.1. Amyloid-β (Aβ) Models

Gain-of-toxicity models of Aβ has been applied to different tissues in *C. elegans*. When expressing human Aβ1-42 in body wall muscles, intriguingly, a mass spectrometry analysis detected the presence of truncated Aβ3-42, rather than the intended full-length Aβ1-42 [93]. Nevertheless, worms accumulated toxic Aβ aggregates and led to progressive paralysis [93,94]. McColl et al. [95] successfully promoted the expression of full-length Aβ1-42 in *C. elegans* muscle cells by an additional insertion of Asp-Ala (DA) to the N-terminus of the human Aβ sequence. These worms showed characteristic degenerative features like soluble Aβ oligomers and behavioural deficits, leading to severe paralysis [95]. Other similar studies confirmed the same observations, and an increased ROS level and decreased lifespan were also noticed [96,97].

Models introducing Aβ into the nervous system of *C. elegans* have been constructed as well. The overexpression of Aβ1-42 in glutamatergic neurons or pan-neurons caused Aβ deposits, neuronal degeneration, behavioural defects and a shortened lifespan. Fluorescence lifetime imaging revealed that Aβ aggregation starts in a subset of neurons and spreads to other tissue during ageing. The RNAi-mediated depletion of Aβ specifically in these neurons effectively delays Aβ aggregation and pathology [98]. Other molecules, such as the transcription factor SPR-4, have also been reported to act as mitigating factors for Aβ-related toxicity [99]. Additionally, an inducible global secretion of Aβ1-42 proteins was constructed to study the time-lapse changes of protein aggregates. Aβ proteins were observed to outspread from neurons and form distinct immobile aggregates extracellularly [100]. Based on the model, a disintegrin and metalloprotease 2 (ADM2) were identified to be capable of removing extracellular Aβ aggregates [100].

Various genetic and drug screenings have been conducted in *C. elegans* Aβ models. An RNAi screen in a muscle expression model revealed that the inhibition of mitoferrin-1 diminished mitochondrial ROS levels, resulting in a reduced paralysis rate and prolonged worm lifespan [101]. Natural products such as *Holothuria scabra* extracts, *Radix Stellariae* extracts and D-pintol have been found to reduce Aβ aggregation and decrease ROS levels in Aβ disease models [96,102,103].

### 4.2. Tau Models

A *C. elegans* homologue of human tau, *ptl-1*, is involved in the maintenance of neural health during ageing [104]. As the loss of its function cannot be fully restored by human tau, the majority of tau models in *C. elegans* opt for the direct expression of human tau and its disease-related variants [104]. The overexpression of disease-associated tau (P301L, V337M and R406W) in the *C. elegans* nervous system caused insoluble tau accumulation and defects in sensory and motor neuronal functions [105,106]. These worms also developed age-dependent breaks in nerve cords following substantial neuronal loss, indicating possible neurodegeneration [105,106]. The overexpression of human HSP70 managed to alleviate the neural dysfunction in these models [107]. A genome-wide RNAi screen employed a pan-neuronal expression model of tau (V337M) and identified 75 genes that aggravated tau (V337M)-induced toxicity. Forty-six of them shared sequential similarities with the human genome, including chaperones and proteases that are part of the proteostasis network [107].

Tau aggregation-mediated toxicity was further supported by introducing pro-aggregation and anti-aggregation mutations in *C. elegans* models [108]. Pro-aggregation mutation with K280 deletion enhanced tau aggregation propensity, while anti-aggregation mutations with a combination of the I277P and I308P mutations prevented β-sheet formation and subsequent aggregation [108]. Worms with pro-aggregation mutations showed impaired mitochondrial transport, severely compromised motility and obvious neuronal dysfunction in comparison to the anti-aggregation combination [108]. The overexpression of another tau-aggregation-prone variant (3PO) also caused the formation of insoluble aggregates and a shortened lifespan [109]. The pan-neuronal overexpression of another disease-associated mutation, tau (V363A/V363I), further differentiated the toxicity of insoluble tau and soluble oligomers. Worms with tau (V363A) formed soluble oligomeric assemblies, while tau (V363I) accumulated as highly phosphorylated insoluble tau assemblies. Interestingly, tau (V363A) impaired presynaptic function in both motor and pharyngeal neurons. In contrast, tau (V363I) only affects postsynaptic function in motor neurons [106].

Consistent tau-induced neurotoxicity has been demonstrated in a single-copy gene insertion model. Two strains were constructed to mimic common post-translational modifications contributing to tauopathy, tau (T231E) for phosphorylation and tau (K274/281Q) for lysine acetylation [110]. Both strains exhibited reduced touch sensation and an abnormal neuronal morphology, while tau (K274/281Q) hampered neuronal mitophagy under mitochondrial stress [110].

Recently, studies have indicated a novel aggregation-independent mechanism of tau toxicity. The overexpression of tau (A152T) in the nervous system leads to severe locomotor defects and gaps in nerve cords, implying motor neuron degeneration [111]. A close inspection of the touch sensory neurons revealed morphological abnormalities such as convoluted neuronal processes and nonspecific outbranching, resembling common characteristics of aged neurons [111]. These worms also showed the aberrant localisation of presynaptic components and neurotransmission defects, as well as an abnormal mitochondrial distribution and trafficking [111]. Strikingly, no insoluble tau aggregate was detected, and the addition of anti-aggregation compounds failed to rescue tau (A152T)-related toxicity [111]. Another piece of evidence is from a pseudo-hyperphosphorylation (PHP) tau model, which overexpressed mutated tau (ten serine/threonine residues to glutamic acid) in *C. elegans*, to mimic the pseudo-hyperphosphorylation status [112]. These worms showed defects in motor neuron development and ageing-related neurodegeneration, but, surprisingly, lacked apparent tau aggregates [112]. Similarly, a model with tau (R406W) expressed in all neurons showed aberrantly phosphorylated tau but no detergent-insoluble aggregates [113]. Drug screening using the same model identified curcumin, a major phytochemical compound in turmeric, that reduced tau-induced toxicity [113].

## 5. Parkinson’s Disease (PD)

Parkinson’s disease (PD) is a neurodegenerative disease characterised by the loss of dopaminergic neurons in the substantia nigra. Patients with PD develop motor symptoms including muscle stiffness, slowness of movement and postural instability, and non-motor symptoms such as sleeping disorder, cognitive impairment and neuronal dysfunction [114,115]. At the cellular level, α-synuclein (αSyn, encoded by the *SNCA* gene) aggregation is considered as the pathological hallmark of PD. In addition, increased ROS levels and impaired autophagy and mitochondria together contribute to PD pathology [116,117,118]. A number of genes were identified to associate with familial forms of PD, including *SNCA*, *LRR*2, *PINK*1 and *PARK*7 [119]. Mutations in these genes either directly lead to abnormal αSyn amyloid fibrils or interfere with the physiological pathways involved in mitochondria and autophagy [115,119,120,121,122].

Despite the lack of a functional orthologue for human αSyn in the *C. elegans* genome, the overexpression of disease-associated mutant SNCA (A53T/A56P/A30P) proteins in *C. elegans* dopamine neurons leads to αSyn accumulation and locomotory defects, therefore phenocopying the cellular and physiological defects described in mammalian PD models [123,124,125,126,127]. Worms with the overexpression of WT human αSyn:Venus fusion in dopamine neurons developed inclusions in the axons and pathological blebbing in the dendrites [128]. The rounded cell bodies and dendritic disorganisation indicated that the process of neurodegeneration was associated with ageing [128]. These worms showed defects in foraging behaviour and the crawling to swimming switch, similar to that induced by dopamine deficiency [128]. Based on this model, reverse genetic screening of >100 PD susceptibility genes identified in a preliminary genome-wide association study (GWAS) yielded 28 genetic modifiers participating in pathways such as calcium signalling and vesicular trafficking [128]. The inactivation of these genes altered the pathological phenotype and alleviated αSyn toxicity [128].

Other studies have been undertaken to introduce human αSyn into different *C. elegans* tissues. The overexpression of WT human SNCA proteins, either strictly in motor or mechanosensory neurons, or broadly in all neurons or the musculature, resulted in the formation of mobile and immobile aggregates and movement defects, indicating an apparent gain-of-toxicity [123,125,129]. RNAi screens using these models have uncovered different cellular pathways that can suppress αSyn-mediated inclusions and modulate neurotoxicity, including histone modification, choline phosphorylation, cytoskeletal components and vesicular endocytosis [125,129]. Other suppressors, *sir-2.1/*SIRT1 and *lagr-1/*LASS2, participate in an ageing-associated cellular pathway, suggesting a potential linkage between αSyn inclusion formation and cellular ageing [130]. A recent high-throughput kinetic screening identified a small molecule, SynuClean-D, as an αSyn aggregation inhibitor in vitro [131]. The treatment of SynuClean-D in *C. elegans* expressing αSyn in both dopaminergic neurons and muscle cells showed substantially reduced proteotoxicity [131]. Furthermore, natural products such as squalamine and chrysin have also been found to suppress αSyn aggregation and alleviate locomotory defects [124,132].

## 6. Conclusions

*C. elegans* has established itself as a favoured model organism in the field of ageing-related disease research. Abundant analyses of gene mutations pertinent to neurodegenerative diseases have been undertaken using this small and simple nematode, recapitulating critical phenotypic features of the diseases. Through these models, *C. elegans* acts as an informative intermediary to provide mammalian studies with novel candidates to probe the complexities of neurodegenerative diseases. Another compelling advantage is the practicability of conducting large-scale high-throughput in vivo drug screenings in *C. elegans* models, where several compounds have been tested for their efficacy against neurotoxicity. The intricacy or simplicity of *C. elegans* does have its drawbacks. The complex and heterogeneous nature of neurodegenerative diseases is difficult to mimic in the simple architecture of the *C. elegans* nervous system. In the context of neurons, the intricately interconnected clusters of neurons, the caudate and putamen, are absent in *C. elegans*, which are the most affected structures in HD patients. In the context of neuronal processes, the absence of myelin sheaths wrapping *C. elegans* axons failed to recapitulate myelin in the human nervous system, the dysfunction of which plays an imperative role in the pathogenesis of neurodegenerative diseases [133]. Moreover, *C. elegans* lacks an adaptive immune system and fails to incorporate and resemble the comorbidities, such as neuroinflammation, that underlie the pathology of these diseases [2]. In addition, to what extent the drug candidates discovered in *C. elegans* models can retain their high efficacy in the human system, or whether they are relevant to human pathology, remains unknown. Nevertheless, these models present novel therapeutic candidates as promising alternatives to the limited effective therapies available currently, and increasing research is being conducted to validate the potency of these drugs in mammalian systems. The worm itself still serves as a robust preclinical tool to enhance our understanding towards the fundamental pathophysiology of neurodegenerative diseases at the molecular and genetic levels. More research is warranted to accelerate this process, potentially by focusing on conserved signalling pathways or molecules involved in disease pathogenesis, which will possibly shed more light on promising disease intervention strategies.

## Data Availability

No new data were created or analyzed in this study. Data sharing is not applicable to this article.
